# Identification of an Alarm Pheromone-Binding Chemosensory Protein From the Invasive Sycamore Lace Bug *Corythucha ciliata* (Say)

**DOI:** 10.3389/fphys.2018.00354

**Published:** 2018-04-06

**Authors:** Fengqi Li, Ningning Fu, Du Li, Hetang Chang, Cheng Qu, Ran Wang, Yihua Xu, Chen Luo

**Affiliations:** ^1^Institute of Plant and Environment Protection, Beijing Academy of Agriculture and Forestry Sciences, Beijing, China; ^2^Stowers Institute for Medical Research, Kansas City, MO, United States

**Keywords:** alarm pheromone, sycamore lace bug, chemosensory proteins, behavioral study, geraniol

## Abstract

The spread of the exotic insect pest sycamore lace bug *Corythucha ciliata* (Say) is increasing worldwide. The identification of behaviorally active compounds is crucial for reducing the current distribution of this pest. In this study, we identified and documented the expression profiles of genes encoding chemosensory proteins (CSPs) in the sycamore lace bug to identify CSPs that bind to the alarm pheromone geraniol. One CSP (*CcilCSP2*) that was highly expressed in nymph antennae was found to bind geraniol with high affinity. This finding was confirmed by fluorescence competitive binding assays. We further discovered one candidate chemical, phenyl benzoate, that bound to CcilCSP2 with even higher affinity than geraniol. Behavioral assays revealed that phenyl benzoate, similar to geraniol, significantly repelled sycamore lace bug nymphs but had no activity toward adults. This study has revealed a novel repellent compound involved in behavioral regulation. And, our findings will be beneficial for understanding the olfactory recognition mechanism of sycamore lace bug and developing a push-pull system to manage this pest in the future.

## Introduction

Sycamore lace bug, *Corythucha ciliata* (Say) (Heteroptera: Tingidae), is an important exotic invasive pest. This insect has spread to many countries and seriously affected city landscapes and disturbed people's lives (Halbert and Meeker, 1983; Wang et al., 2008; Ju et al., 2015). Currently, the management of this pest is dependent on insecticides, such as pyridines (Ju et al., 2015). The discovery of behaviorally active compounds is an efficient approach to developing pest management strategies (Foster and Harris, 1997). Examples of these types of compounds include alarm pheromones, which affect insect behavior, development and oviposition and are important for insects to defense against dangers such as natural enemies (Ono et al., 2003; Kunert et al., 2005; Dewhirst et al., 2010). In addition, alarm pheromones and other repellent compounds can enhance the efficiency of insecticides (Kuwahara et al., 2011). Thus, these types of compounds are important targets in pest management. The alarm pheromone of sycamore lace bug has been identified as geraniol, *E*-3,7-dimethyl-2,6-octadien-1-ol (Kuwahara et al., 2011). Geraniol is only detected in the nymphs of this pest, indicating that it does not act on adults (Kuwahara et al., 2011). In nymphs, geraniol acts a repellent. For example, sycamore lace bug nymphs crowd together on the leaves of platanus trees and show evasive behavior when an individual in the center of the crowd is squashed and emits geraniol (Kuwahara et al., 2011). Interestingly, geraniol is also the alarm pheromone of the chrysanthemum lace bug, *Corythucha marmorata*, which is a new exotic invasive pest in Asia (Watanabe and Shimizu, 2015), and one of the alarm pheromones in the hawthorn lace bug, *Corythucha cydoniae*, and eggplant lace bug, *Gargaphia solani* (Aldrich et al., 1991). Thus, geraniol may be common to insect species in Tingidae. Although geraniol may potentially be used to control sycamore lace bugs and multiple other insect species, it is unstable in the environment, and this limits its large-scale application.

Chemosensory proteins (CSPs) are a class of soluble carrier proteins that are thought to be involved in insect chemoreception (Kulmuni and Havukainen, 2013), and each Hemipteran insect has approximately 10 CSP genes (Zhou et al., 2010; Gu et al., 2013; Wu et al., 2016; Wang et al., 2017). Based on three-dimensional (3D) structures, CSPs consist of 6 alpha helices stabilized by α-α loops. There are four highly conserved and structurally important cysteines that are connected by two pairs of non-interlocked disulphide bridges (Wanner et al., 2004).

CSPs are thought to be associated with chemoreceptive sense to chemical information in insect. Research on CSPs can promote the development of insect behaviorally active compounds. However, at present, we lack of understanding of the CSPs in this pest. In the current study, we combined bioinformatics analysis, expression profiling, molecular docking, fluorescence competitive binding assays and behavioral studies to identify the sycamore lace bug CSPs that recognize geraniol. We also performed a virtual screen for additional compounds that potentially bound to CSPs and then determined which of these compounds could significantly regulate the behavior of this pest. The novel insect behavior control compounds that we have identified in this study will improve our understanding of the olfactory mechanisms of sycamore lace bug and facilitate the development of strategies to control the behavior of this important pest.

## Materials and methods

### Insects

Sycamore lace bugs were obtained from *Platanus* × *acerifolia* trees grown at the Chinese Academy of Agricultural Science, Haidian District, Beijing. A population of sycamore lace bugs was reared on *P*. × *acerifolia* leaves in a greenhouse under the following conditions: temperature 25 ± 2°C, humidity 50–70%, and a 16L−8D photoperiod. The nymphs and adults were collected from the leaves for expression analysis and behavioral studies.

### Identification and expression pattern of CSPs

Our previously published transcriptome datasets were used in this study (Li et al., 2016, 2017). Briefly, total RNA was extracted from sycamore lace bugs at different developmental stages (nymphs, female adults, and male adults) and physiological stages (dormant and non-dormant) using the RNAqueous-Micro kit (Life Technologies, Carlsbad, CA, USA). From these RNA samples, cDNA libraries were constructed and sequenced using the Illumina HiSeq2500 sequencer at Novogene Company (Beijing, China). Sequencing data were deposited into the NCBI Sequence Read Archive (SRA) under accession numbers SRR3170921, SRR3170922, SRR3170923, SRR3883369, and SRR3883370. To identify CSPs, known hemipteran CSP amino acid sequences were used as queries in tBLASTn searches against the transcriptome database. Then, the putative CSPs were confirmed by BLASTx searches against the nr database in NCBI (*E* < 1.0E-5).

RNA for expression analysis of individual genes was extracted from sycamore lace bugs using the same method as for the transcriptome analysis. The quantity of the total RNA was determined using DS-11 Spectrophotometer (DeNovix, USA). The cDNA was synthesized from 1 μg of total RNA using PrimeScript RT Master Mix (Perfect Real Time) (TaKaRa, Dalian, China). Quantitative Real-Time PCR (qRT-PCR) was carried out to determine the expression patterns of sycamore lace bug CSPs in the antennae of nymphs, adult males and adult females and the transcript levels of *CcilCSP2* in different nymph tissues. All gene-specific primers were designed using the Integrated DNA Technologies web site (http://sg.idtdna.com). Primer sequences are listed in Table S1. GAPDH (Genbank number: MG948453) and 18S rRNA(Genbank number: MG948452) were used as internal controls. The 2 × GoTaq® qPCR Master Mix (Promega, Madison, WI, USA) was used for qRT-PCR performed on an ABI Prism®7500 (Applied Biosystems, Carlsbad, CA, USA). The PCR-cycling conditions were as follows: 95°C for 2 min, 40 cycles of 95°C for 30 s, 60°C for 1 min, and a final melting cycle at 95°C for 15 s, 60°C for 15 s, 95°C for 15 s. For each sycamore lace bug CSP, qRT-PCR was repeated three times on three independent biological replicates. Relative expression levels of genes were calculated using the 2^−ΔΔCt^ method (Pfaffl and, 2001).

### Cloning of CSP genes

To clone the CSP genes, gene-specific primers were designed using Primer version 5.0 (Lalitha, 2004). PCR amplification was performed using the PrimeSTAR HS DNA Polymerase (Takara, Dalian, China) with the following conditions: 95°C for 1 min; 30 cycles of 98°C for 10 s, 58°C for 15 s, and 72°C for 1 min, and a final extension at 72°C for 10 min. The PCR products were purified with the EasyPure Quick Gel Extraction Kit (TransGen, Beijing, China). Then, the fragments were ligated into the pEASY-Blunt cloning vector (TransGen, Beijing, China) and sequenced by TsingKe (Beijing, China). The protein coding regions were predicted with ORF Finder (http://www.ncbi.nlm.nih.gov/projects/gorf/). The phylogenetic relationships were determined using the neighbor-joining algorithm with 1,000 bootstrap replicates.

### Molecular docking

We searched for potential templates for CcilCSP2 in the NCBI Protein Data Bank (PDB) database using the BLAST server. The homology modeling of CcilCSP2 was performed using the SWISS-MODEL function in Swiss Pdb viewer. The model was further refined by molecular dynamics simulations.

The final 3D model was assessed using several methods on the online Structure Analysis and Verification Server (http://services.mbi.ucla.edu/SAVES/), including Procheck, Verify_3D and ERRAT. The evaluation of PDF total energy was carried out on the ProSA-web server (Wiederstein and Sippl, 2007). The best models for CcilCSP2 were confirmed using the evaluation of PDF total energy, verify score and Ramachandran plots.

A total of 101 compounds were downloaded from the ZINC database. These compounds included commercially available host volatile substances, insect pheromones or their analogs (Table S2). The virtual screen was performed using AutoDock Vina and AutoDock. First, the AutoDock Vina program was used to perform automated computational docking to quickly obtain docking scores for the binding of these compounds with CSPs. Then, the binding modes of compounds with docking scores <-7 were further estimated using AutoDock version 4.2. The Lamarckian genetic algorithm was used for molecular docking; 100 Lamarkian genetic algorithm runs were performed with 25 × 10^6^ evaluations.

### Fluorescence competitive binding assays

CcilCSP2 was expressed and purified following our previously published protocols (Chang et al., 2015). Briefly, the sequence of CcilCSP2 encoding the mature CSP protein was expressed in *Escherichia coli* BL21 (DE3) competent cells (Transgen). Cells were grown at 37°C until OD600 ≈ 0.60, when expression was induced with 1 mM IPTG. After incubating an additional 8 h at 28°C, cells were harvested by centrifugation. The purification of CcilCSP2 was performed using HisTrap affinity columns (GE Healthcare Biosciences, Uppsala, Sweden). To measure the binding affinity of CcilCSP2 to the fluorescent probe N-Phenyl-1-naphthylamine (1-NPN), a 2 μM protein solution in Tris-HCL buffer (pH 7.4, 50 mM) was titrated with 1 mM 1-NPN in methanol to a final concentration of 2–16 μM. The binding of each ligand was tested in competitive binding assays using 1-NPN as the fluorescent reporter and final concentrations of 0.2–1.6 μM for each ligand. Binding constants of competitors were calculated from the corresponding IC50 values using the following equation: *K*_*D*_ = [IC50]/1+[1-NPN]/*K*_1−*NPN*_), with [1-NPN] being the free concentration of 1-NPN and *K*_1−*NPN*_ being the binding constant of the protein complex/1-NPN.

### Behavioral study of sycamore lace bug adults

Compounds that potentially bind with high affinity to CSPs were purchased from Sigma (St Louis, MO, USA) to test their effects on sycamore lace bug behavior. The behavioral response of sycamore lace bug adults (1:1 sex ratio) was assessed using a Y-shaped olfactometer. The length of the arms and the diameter of the tube were 10 and 5 cm, respectively. The authentic standards were dissolved in hexane at concentrations of 1 and 0.1 μg/μl. Then 10 μl of solution was applied to a 1 cm^2^ filter paper. The filter paper was placed in one arm of the Y-shaped olfactometer, and 10 μl hexane was placed in the other arm as a control. The amount of airflow was set at 250 ml/min. In each experiment, a single sycamore lace bug adult was released at the base of the olfactometer stem and observed for 10 min. During this time, insects not making any choice were recorded as having “no response”. Insects entering more than halfway into the olfactometer arm and staying for at least 10 s were recorded as having a “response”. After 10 insects were tested, the orientation of the Y-shaped olfactometer was reversed to avoid environmental effects, and the inner wall was cleaned with degreased cotton containing acetone, washed with ethanol, and then washed with distilled water and dried in an oven. The experiment was conducted in a behavioral observation chamber at 26°C. The experiment was repeated seven times, and each time the behavior of 10 insects was tested. The choice of insects between chemicals and hexane was compared using chi-square analysis.

### Behavioral study of sycamore lace bug nymphs

The Y-shaped olfactometer strategy is not suitable for studying the behavior of sycamore lace bug nymphs due to their small body size. Therefore, two alternative methods were used for tests of nymph behavior.

#### Petri dish test

One test was conducted in a petri dish (15 cm diameter) at 22–26°C. Two filter papers (3 cm diameter) were placed symmetrically on both sides of the petri dish. 20 μl of the compound to be tested (1, 0.1 or 0.01 μg/μl) was added to one filter paper. 20 μl hexane was added as a control to the other filter paper. The filter papers were placed at a distance of 5 cm from the start position at the center of the petri dish where 40 sycamore lace bug nymphs were released. The number of nymphs on each filter paper was recorded after 5 min. Each assay was replicated four times with a new petri dish for each replicate. The choice between hexane and a blank was also tested. The choices between those compounds that were found to affect nymph behavior and geraniol were also tested. The numbers of insect on each filter paper were compared using chi-square analysis.

#### Plant leaf test

To further confirm the behavioral effects of each active substance, the behavioral response of sycamore lace bug nymphs was tested by directly applying the compounds to a *P*. × *acerifolia* leaf. One microliter tested compound (1 or 0.1 μg/μl) was applied at a distance of 0.5 mm from a squashed colony. Photographs were then taken at 0, 2, and 4 min to assess the repellency rate, which was calculated using the following formula: Repellency rate = M/T^*^100, where T = the total number of sycamore lace bug nymphs in the tested colony at 0 min, and M = the number of sycamore lace bug nymphs that had moved at 2 or 4 min. Experimental conditions were the same as for the petri dish experiments. Hexane was used as the mock treatment. The repellency rates of the nymphs in each treatment were compared using a Kruskal Wallis H-test followed by Mann-Whitney U-tests.

## Results

### The behavioral response of sycamore lace bug to geraniol

To determine if adults and nymphs respond differently to geraniol, we performed behavioral assays. Behavior was assayed in a Y-shaped olfactometer for adults and in petri dishes and on leaves for nymphs. Sycamore lace bug adults and nymphs had different behavioral responses to geraniol. The nymphs were significantly repelled by geraniol in petri dishes (Table 1) and on plant leaves (**Table 5**). However, the behavior of sycamore lace bug adults was not significantly affected by geraniol (Y-tube, 1 μg/μl geraniol: χ^2^ = 0.069, *P* > 0.05; 0.1 μg/μl geraniol: χ^2^ = 1.52, *P* > 0.05).

**Table 1:** The behavioral response of sycamore lace bug nymphs to geraniol and phenyl benzoate in petri dish tests.

	**0.01 μg/μl**	**0.1 μg/μl**	**1 μg/μl**
Geraniol vs. Hexane	5.444^a^^*^	10.965^**^	13.928^**^
Phenyl benzoate vs. Hexane	0.615	14.629^**^	5.818^*^
Geraniol vs. Phenyl benzoate	0.214	0.267	0.083
Naphthalene vs. Hexane	0.243	1.190	0.333
p-Cymene vs. Hexane	0.083	0.026	0.048
Thymol vs. Hexane	0.400	0.111	0.125
Hexane vs. Blank	0.020	0.160	0.169

a *Chi-square value*.

* *P < 0.05*,

** *P < 0.01*.

### The CSPs involved in binding geraniol

**Figure 1:**
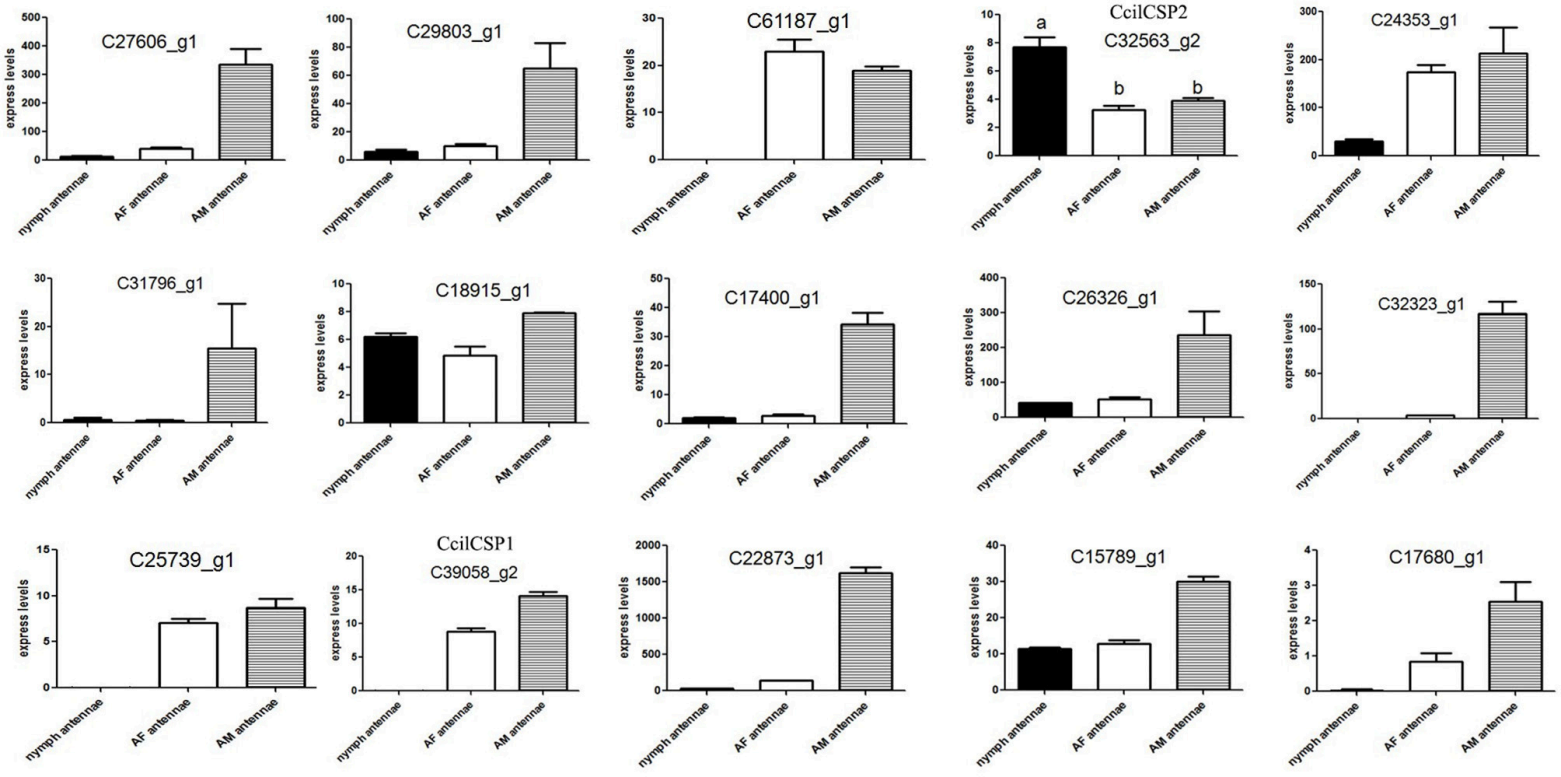
Relative expression levels of candidate CSP genes in the antennae of sycamore lace bugs. AF, adult female; AM, adult male. Transcription levels of the *CcilCSP2* gene were normalized by GAPDH and the 18S rRNA gene. Data are presented as the mean (± SD), and different letters indicate significant differences in transcript levels (*p* < 0.05, LSD test).

**Figure 2:**
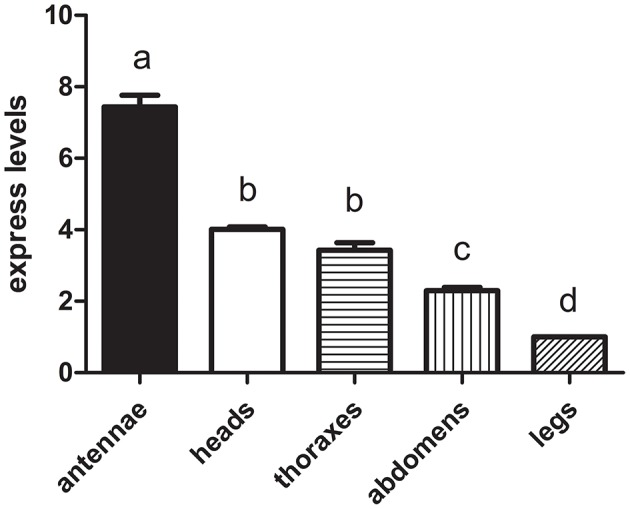
The transcript levels of *CcilCSP2* in different nymph tissues. Transcription levels of the *CcilCSP2* gene were normalized by GAPDH and the 18S rRNA genes. Transcript levels are shown relative to those in the leg. Data are presented as the mean (± SD), and different letters indicate significant differences in transcript levels (*p* < 0.05, LSD test).

**Figure 3:**
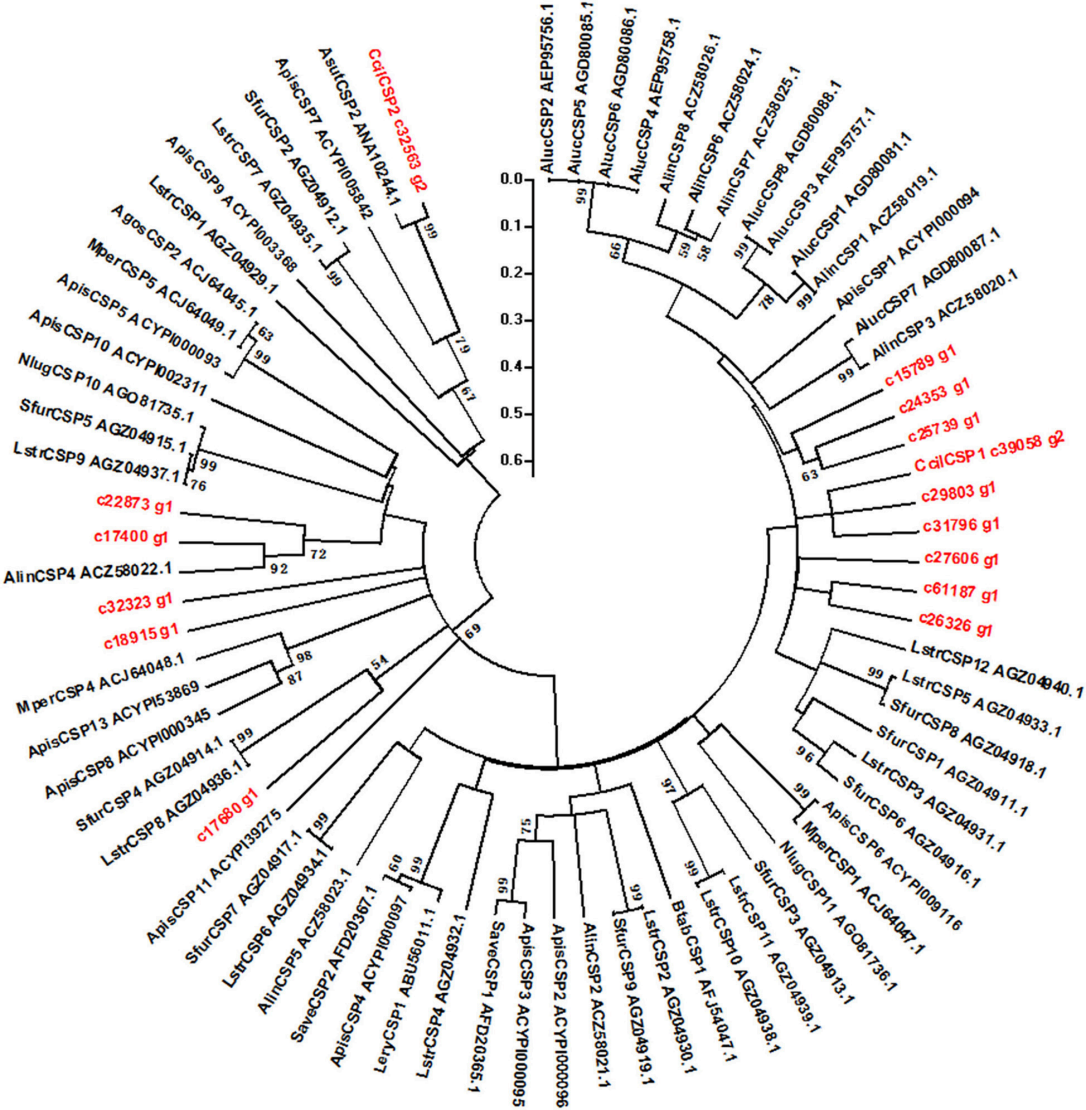
Phylogenetic tree of CcilCSP2 and related CSPs from other insects. Only bootstrap values > 50% are shown. The scale bar indicates the evolutionary distances.

We hypothesized that the CSPs involved in the perception of geraniol would be more highly expressed in the antennae of nymphs than in the antennae of adult males and females. To test this hypothesis, a total of 15 CSPs were identified from the sycamore lace bug RNA-seq datasets and their expression patterns were compared. These 15 CSPs include one previously reported CcilCSP1 (Fu et al., 2017) and 14 new CSP genes. Only one CSP (c32563_g2) was expressed at a higher level in the antennae of nymphs than in the antennae of adult males and females (Figure 1). Moreover, this CSP was significantly more highly expressed in antennae than other nymph tissues (Figure 2). Thus, the expression levels of c32563_g2 are associated with the differing responses of sycamore lace bug nymphs and adults to geraniol, and this CSP was selected as a candidate geraniol-binding protein. The sequence of c32563_g2 was confirmed by molecular cloning and sequencing, and phylogenetic analysis with known CSPs in other insect species revealed that c32563_g2 clustered with the *Adelphocoris suturalis* Jakovlev CSP2 protein (82% identity at the amino acid level). Based on the nr annotation (Table 2) and phylogenetic relationships (Figure 3), we named c32563_g2 as *CcilCSP2*.

**Table 2:** Candidate sycamore lace bug chemosensory protein unigenes.

**Genes**	**Genbank numbers**	**NR ID**	**NR score**	**NR E-value**	**NR description**	**Full length (AA)**
c27606_g1	MG948438	AGD80088.1	293	6.43E−29	Chemosensory protein 8 (*Apolygus lucorum*)	123
c29803_g1	MG948439	ADG96052.1	142	6E−39	Putative chemosensory binding protein (*Stomoxys calcitrans*)	131
c61187_g1	MG948440	AGD80088.1	315	3.79E−34	Chemosensory protein 8 (*A. lucorum*)	128
CcilCSP2 (c32563_g2)	MG948441	ANA10244.1	187	4E−58	Chemosensory protein 2 (*Adelphocoris suturalis*)	111
c24353_g1	MG948442	AEP95757.1	342	1.76E−36	Chemosensory protein 3 (*A. lucorum*)	130
c31796_g1	MG948443	ACJ64054.1	349	8.87E−38	Putative chemosensory protein CSP8 (*Nilaparvata lugens*)	128
c18915_g1	MG948444	AHX37222.1	195	7.1E−16	Chemosensory protein 8 (*Conogethes punctiferalis*)	133
c17400_g1	MG948445	ACZ58022.1	420	7.9E−50	Chemosensory protein 4 (*Adelphocoris lineolatus*)	131
c26326_g1	MG948446	AEP95757.1	400	3.66E−46	Chemosensory protein 3 (*A. lucorum*)	129
c32323_g1	MG948447	ACZ58022.1	264	9.36E−26	Chemosensory protein 4 (*A. lineolatus*)	134
c25739_g1	MG948448	AEP95755.1	291	4.44E−30	Chemosensory protein 1 (*A. lucorum*)	130
CcilCSP1 (c39058_g2)	KY354042	AEP95755.1	726	0.0	Chemosensory protein 1 (*C. ciliata*)	130
c22873_g1	MG948449	ACZ58022.1	308	1.01E−32	Chemosensory protein 4 (*A. lineolatus*)	125
c15789_g1	MG948450	AGD80084.1	395	2.04E−45	Chemosensory protein 4 (*A. lucorum*)	131
c17680_g1	MG948451	AGZ04936.1	112	2.29E−26	Chemosensory protein 8 (*Laodelphax striatella*)	148

### Docking analysis of CcilCSP2

Using the CcilCSP2 amino acid sequence as a query in a blastp search against the PDB database, three CSP templates, including a CSP from the desert locust *Schistocerca gregaria* (PDB: 2GVS, 29% identity), antennal CSP A6 from *Mamestra brassicae* (PDB: 1KX8, 26% identity), and CSP 1 from *Bombyx mori* (PDB: 2JNT, 26% identity) were selected for homology modeling. After the evaluation of model qualities, the G-factor values (Table 3) were all greater than−0.5, which indicated that the distribution of torsion angles and covalent geometries of the model proteins were reasonable. Greater than 82.42% of the residues had an average 3D-1D score > 0.2 in VERIFY 3D, the overall quality factor was > 60.241 in ERRAT, and the Z-score for CcilCSP2 was−4.96. These parameters indicated that the protein model obtained by homology modeling was reliable.

**Table 3:** CcilCSP2 model quality estimations.

**Gene**	**G-factor**					
	**Dihedrals**	**Covalent**	**Overall**	**Verify_3D**	**ERRAT**	**Z-score**
CcilCSP2	−0.09	−0.46	−0.39	82.42%	60.241	−4.96

In addition to geraniol, four compounds, including naphthalene, thymol, p-Cymene, and phenyl benzoate were identified as potential ligands of CcilCSP2 using AutoDock Vina and then verified by AutoDock4.2. The binding affinities of these five compounds for CcilCSP2 are shown in Table 4. Naphthalene, thymol, p-Cymene, phenyl benzoate and geraniol) exhibited low binding energies (< −5.33) and had inhibition constants (K_i_) < 123 μM. Notably, phenyl benzoate had the lowest binding energies (−6.42) and K_i_ (19.75 μM) (Table 4).

**Table 4:** Binding affinities of six compounds for CcilCSP2 estimated by AutoDock 4.2.

**Name**	**CcilCSP2**		
	**Cas**	**Binding energy**	**k_l_ (μM)**
Geraniol 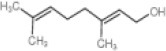	106-24-1	−5.33	122.98
Naphthalene 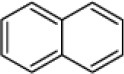	91-20-3	−5.68	68.86
Thymol 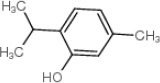	89-83-8	−5.36	117.04
p-Cymene 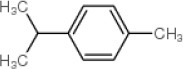	99-87-6	−5.82	54.53
Phenyl benzoate 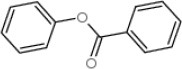	93-99-2	−6.42	19.75

**Figure 4:**
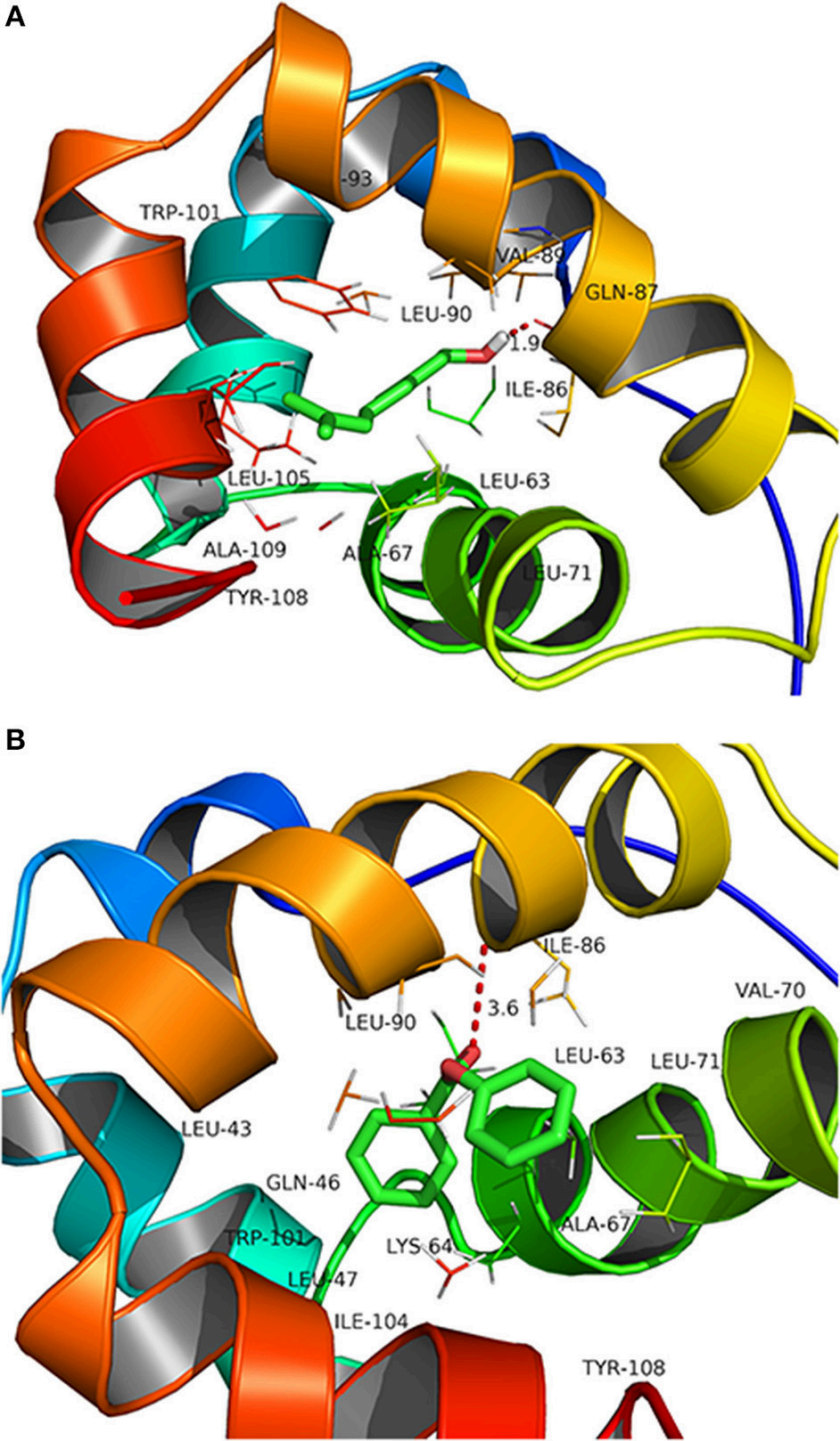
Molecular docking of CcilCSP2 with geraniol **(A)** and phenyl benzoate **(B)**.

Docking analysis showed that CcilCSP2 binds phenyl benzoate and geraniol in the same region, which includes amino acid residues Leu-90, Val-89, Gln-87, Ile-86, Ieu-63, Ala-67, Ala-109, Leu-105, and Trp-101 (Figure 4). Furthermore, CcilCSP2 amino acid residues Ile-86 forms a hydrogen bond with both phenyl benzoate and geraniol (Figure 4).

### CcilCSP2 ligand-binding properties

**Figure 5:**
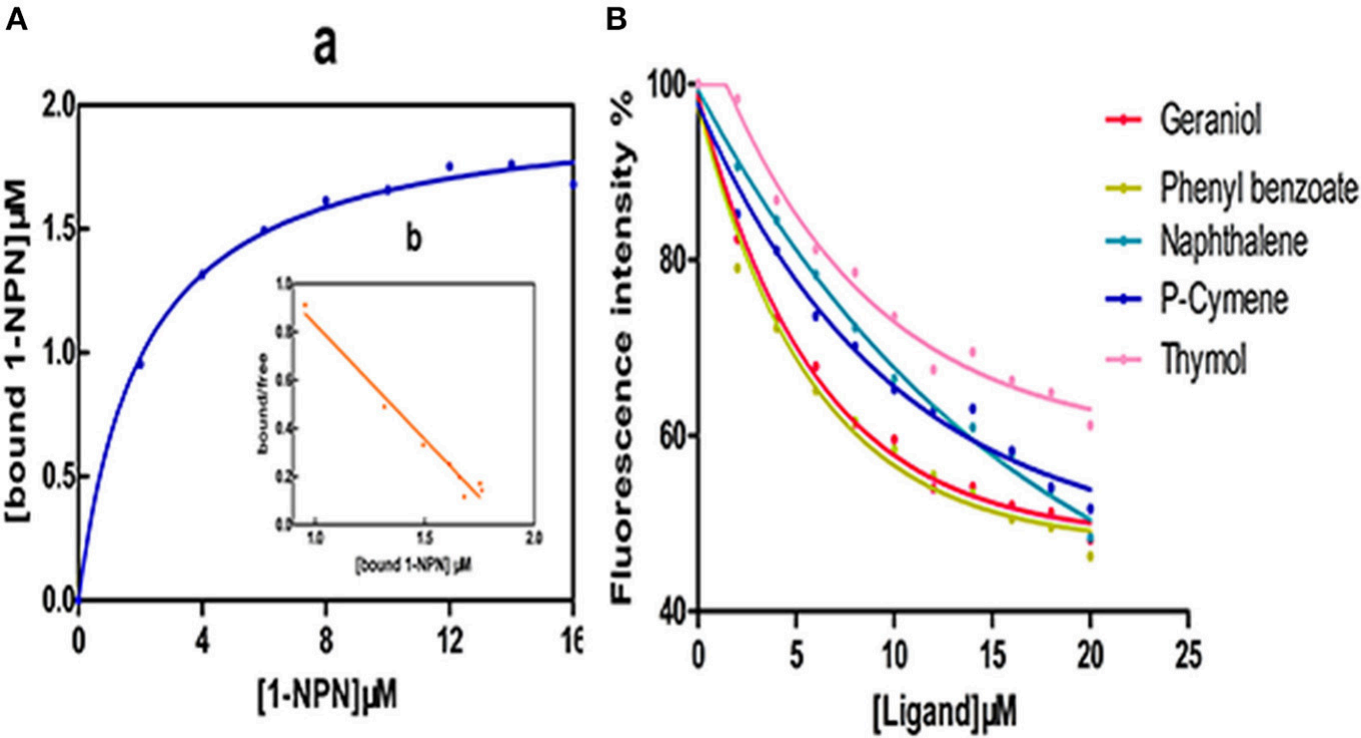
Binding of N-phenyl-1-naphthylamine (1-NPN) and selected ligands to CcilCSP2. **(A)** Affinity of CcilCSP2 for 1-NPN. (a), Binding curve. (b), Scatchard plot. **(B)** Competitive binding assays.

The dissociation constant (K_D_) for CcilCSP2 bound to 1-NPN was 2.081 μM (Figure 5A), and 1-NPN was used as a fluorescent reporter to test the binding affinities of CcilCSP2 to different ligands. Based on the IC50 and K_D_ values calculated from the ligand binding curves (Figure 5B), CcilCSP2 has high binding affinity for geraniol and phenyl benzoate (Displacement of more than 50% of 1- NPN, K_D_ = 15.16 and 13.93 μM, respectively). Other ligands were not able to displace more than 50% of 1- NPN from CcilCSP2. We also tested another CSP (c18915_g1) that is expressed more highly in the antennae of nymphs than in the antennae of adult females. However, this CSP did not bind to any of these five ligands (Figure S1).

### The behavioral response of sycamore lace bug to the four candidate compounds

The behavioral responses of sycamore lace bug nymphs to phenyl benzoate, naphthalene, p-Cymene and thymol were tested using the petri dish test. There was no significant difference in the choice of nymphs between hexane and the blank (Table 1), indicating that hexane does not affecting nymph behavior. Of the four chemicals tested, only phenyl benzoate significantly repelled sycamore lace bug nymphs (Table 1). There was no significant difference in the choice of nymphs between phenyl benzoate and geraniol (Table 1). The repellent effect of phenyl benzoate was further confirmed by plant leaf tests (Table 5), and there was no significant difference between the nymph repellency by phenyl benzoate and geraniol at doses of 1 and 0.1 μg/μl (*P* > 0.05). The behavioral response of adult sycamore lace bugs to phenyl benzoate was also not significant (Y-tube test, 1 μg/μl phenyl benzoate: χ^2^ = 0.176, *P* > 0.05; 0.1 μg/μl phenyl benzoate: χ^2^ = 0.083, *P* > 0.05).

**Table 5:** The repellency rate of sycamore lace bug nymphs to geraniol and phenyl benzoate in plant leaf tests.

		**0.01 μg/μl**	**0.1 μg/μl**	**1 μg/μl**
	**Mock**		**Geraniol**	
2 min	8.42%	25%^*^	50%^*^	72.41%^*^
4 min	8.68%	25%^*^	57.14%^*^	79.41%^*^
	**Mock**		**Phenyl benzoate**	
2 min	8.65%	12%	50%^*^	63.16%^*^
4 min	8.75%	13%	57.14%^*^	73.68%^*^

* *P < 0.05*.

## Discussion

Identification and expression profiling of chemosensory genes are vital for exploring their roles in insect behavior (Calvello et al., 2005; Du et al., 2016; Zhu et al., 2016a,b). To date, there have been no reports on the olfactory mechanisms of sycamore lace bugs. In our study, we found that geraniol only affected the behavioral responses of sycamore lace bug nymphs. This is consistent with the previous detection of geraniol exclusively in nymphs (Kuwahara et al., 2011). This characteristic also provided us a clue for identifying candidate key olfactory genes in this study. We previously identified 15 CSPs by sequencing and analyzing the sycamore lace bug transcriptome data (Li et al., 2016), which is similar to the number of CSPs identified in other Hemipteran species (Zhou et al., 2010, 2014). Of these CSPs, we identified CcilCSP2 as a putative geraniol-binding protein based on a combination of behavioral studies, expression pattern analysis, and fluorescence competitive binding assays. The characteristics of CcilCSP2 reported here will provide benefit for functionalizing the putative homologous gene CSP2 in *A. suturalis* (Table 2 and Figure 3; Cui et al., 2016). Moreover, such expression characteristics allow us to use this model to further investigate the odorant binding proteins and olfactory receptors in *C. ciliata* to identify the alarm pheromone and new repellents.

In addition to geraniol, phenyl benzoate was identified as a ligand of CcilCSP2 and was shown to repel sycamore lace bug nymphs and to disrupt aggregations of nymphs on leaves. Phenyl benzoate is widely used as a starting chemical in the production of polyesters (Rosenfeld, 1987) and has many properties that make it more stable in open-field environments than geraniol, such as resistance to heat and UV irradiation (Gooch, 2011). Phenyl benzoate could thus serve as a behavioral regulation compound in push-pull systems in the future. Although the structure of geraniol and phenyl benzoate are very different, these two chemicals both interact with Ile-86 through hydrogen bonds. The detailed 3D structures should be studied in future.

Interestingly, like geraniol, phenyl benzoate only repels sycamore lace bug nymphs, indicating that this compound may share the same or similar mechanisms as geraniol. Therefore, phenyl benzoate is also a candidate for the behavioral regulation of other Tingidae species whichgeraniol is the alarm pheromone, including chrysanthemum lace bug (*C. marmorata*), hawthorn lace bug (*C. cydoniae*) and eggplant lace bug (*B. solani*) (Aldrich et al., 1991; Watanabe and Shimizu, 2015). The behavioral response of these species to phenyl benzoate needs to be tested in the future.

Molecular docking is an important method to discover new chemicals based on the structure of proteins with known functions. This method has played an important role in drug discovery (Ruan et al., 2013), and some attractants and repellent chemicals for some insect species, including mosquitoes and aphids, have been also identified by this method (Dhivya and Manimegalai, 2014; Qin et al., 2016; Wang et al., 2016). In our previous paper (Fu et al., 2017), we investigated the interaction between CcilCSP1 and host-plant volatiles using molecular docking, and our findings suggested that this method is useful for searching the pheromone substance of sycamore lace bug. In the current study, our identification of a novel repellent compound demonstrates the potential power of molecular docking and subsequent *in vitro*/*in vivo* evaluation for developing chemicals that regulate insect behavior, and discovering olfactory protein-interacting molecules. Furthermore, this study provides guidelines for screening new repellents for sycamore lace bug nymphs. Specifically, potential repellents could be tested by molecular docking and binding experiments with CcilCSP2, and then only chemicals binding CcilCSP2 could be further tested for effects on behavior. Similar methods could also be used to identify repellent compounds in other Tingidae insect species.

The current study increases our understanding of the functions of *CcilCSP2* in sycamore lace bug and the compounds that attract or repel this important pest. In addition, *CcilCSP2* could be targeted by CRISPR/Cas9 or RNAi as a way to control this pest. Furthermore, detailed study of the three-dimensional structure of CcilCSP2 and its interaction with geraniol and phenyl benzoate could help in the design of new compounds that mimic alarm pheromones and affect sycamore lace bug nymph behaviors in the field.

## Author contributions

Conceived and designed the experiments: FL, YX, and CL; Performed the experiments: FL, NF, DL, and HC; Analyzed the data: FL, NF, DL and HC; Contributed reagents, materials, analysis tools: CQ and RW; Wrote the paper: FL and CL.

### Conflict of interest statement

The authors declare that the research was conducted in the absence of any commercial or financial relationships that could be construed as a potential conflict of interest.

## Supplementary material

Figure S1 Binding of N-phenyl-1-naphthylamine (1-NPN) and selected ligands to c18915_g1. **(A)** Affinity of c18915_g1 for 1-NPN. **(B)** Competitive binding assays.

Table S1 Primers used in this study.

Table S2 Chemicals used in molecular docking.

## References

[B1] Aldrich J. R. Neal J. W. Oliver J. E. Lusby W. R. (1991). Chemistryvis-à-vis maternalism in lace bugs (*Heteroptera: Tingidae*): alarm pheromones and exudate defense in *Corythucha* and *Gargaphia* species. J. Chem. Ecol. 17, 2307–2322. 10.1007/BF00988010 24258608

[B2] Calvello M. Brandazza A. Navarrini A. Dani F. R. Turillazzi S. Felicioli A.. (2005). Expression of odorant-binding proteins and chemosensory proteins in some *Hymenoptera*. Insect Biochem. Mol. Biol. 35, 297–307. 10.1016/j.ibmb.2005.01.002 15763466

[B3] Chang H. Liu Y. Yang T. Pelosi P. Dong S. Wang G. (2015). Pheromone binding proteins enhance the sensitivity of olfactory receptors to sex pheromones in *Chilo suppressalis*. Sci. Rep. 5:13093. 10.1038/srep13093 26310773 PMC4550830

[B4] Cui H. H. Gu S. H. Zhu X. Q. Wei Y. Liu H. W. Khalid H. D.. (2016). Odorant-binding and chemosensory proteins identified in the antennal transcriptome of *Adelphocoris suturalis* Jakovlev. Comp. Biochem. Physiol. Part D Genom. Proteom. 24, 139–145. 10.1016/j.cbd.2016.03.001 27085212

[B5] Dewhirst S. Y. Pickett J. A. Hardie J. (2010). Aphid pheromones. Vitamin Horm. 83, 551–574. 10.1016/S0083-6729(10)83022-5 20831961

[B6] Dhivya R. Manimegalai K. (2014). *In silico* molecular docking and molecular dynamics applications in the designing of a new mosquito repellent from the plant *Calotropis gigantea* targeting the odorant binding protein of *Culex quinquefasciatus*. Int. J. Pharm. Phytopharmacol. Res. 3, 134–138.

[B7] Du L. X. Liu Y. Wang G. R. (2016). Molecular mechanisms of signal transduction in the peripheral olfactory system of insects. Sci. Sin. 46, 573–583. 10.1360/N052016-00163

[B8] Fu N. N. Liu J. Qu C. Wang R. Xu Y. Luo C. (2017). Analysis of *Corythucha ciliata* CcilCSP1 structure and prediction of its binding to host-plant volatiles. Sci. Silvae Sini. 53, 109–117.

[B9] Foster S. P. Harris M. O. (1997). Behavioral manipulation methods for insect pest-management. Ann. Rev. Entomol. 42:123. 10.1146/annurev.ento.42.1.123 15012310

[B10] Gu S. H. Wu K. M. Guo Y. Y. Field L. M. Pickett J. A. Zhang Y. J.. (2013). Identification and expression profiling of odorant binding proteins and chemosensory proteins between two wingless morphs and a winged morph of the cotton aphid *Aphis gossypii* glover. PLoS ONE 8:e73524. 10.1371/journal.pone.0073524 24073197 PMC3779235

[B11] Gooch J. W. (2011). Phenyl Benzoate. New York, NY: Springer

[B12] Halbert S. E. Meeker J. R. (1983). The sycamore lace bug, *Corythucha ciliata* (Say) (*Hemiptera: Tingidae*). Ann. Entomol. Soc. Am. 76, 262–265.

[B13] Ju R. T. Li Y. Z. Feng W. Du Y. Z. (2015). Spread of and damage by an exotic lacebug, *Corythuca ciliata* (Say, 1832) (*Hemiptera: Tingidae*), in China. Entomol. News 120, 409–414. 10.3157/021.120.0410

[B14] Kulmuni J. Havukainen H. (2013). Insights into the evolution of the CSP gene family through the integration of evolutionary analysis and comparative protein modeling. PLoS ONE 8:e63688. 10.1371/journal.pone.0063688 23723994 PMC3665776

[B15] Kunert G. Otto S. Röse U. S. R. Gershenzon J. Weisser W. W. (2005). Alarm pheromone mediates production of winged dispersal morphs in aphids. Ecol. Lett. 8, 596–603. 10.1111/j.1461-0248.2005.00754.x

[B16] Kuwahara Y. Kawai A. Shimizu N. Tokumaru S. Ueyama H. (2011). Geraniol, *E*-3,7-dimethyl-2,6-octadien-1-ol, as the alarm pheromone of the sycamore lace bug *Corythucha ciliata* (Say). J. Chem. Ecol. 37, 1211–1215. 10.1007/s10886-011-0025-2 22076683

[B17] Lalitha S. (2004). Primer Premier 5. Biotech Software & Internet Report, 1, 270–272.

[B18] Li F. Q. Wang R. Qu C. Fu N. Luo C. Xu Y. (2016). Sequencing and characterization of the invasive sycamore lace bug *Corythucha ciliata* (*Hemiptera: Tingidae*) transcriptome. PLoS ONE 11:e0160609. 10.1371/journal.pone.0160609 27494615 PMC4975459

[B19] Li F. Q. Fu N. N. Qu C. Wang R. Xu Y. H. Luo C. (2017). Understanding the mechanisms of dormancy in an invasive alien sycamore lace bug, *Corythucha ciliata* through transcript and metabolite profiling. Sci. Rep. 7:2631. 10.1038/s41598-017-02876-w 28572631 PMC5453966

[B20] Ono M. Terabe H. Hori H. Sasaki M. (2003). Insect signalling: components of giant hornet alarm pheromone. Nature 424, 637–638. 10.1038/424637a 12904781

[B21] Pfaffl M. W. (2001). A new mathematical model for relative quantification in real-time RT–PCR. Nucleic Acids Res. 29, e45–e45. 10.1093/nar/29.9.e45 11328886 PMC55695

[B22] Qin Y. G. Zhang J. P. Song D. L. Duan H. X. Li W. H. Yang X. L. (2016). Novel (*E*)-β-farnesene analogues containing 2-nitroiminohexahydro-1,3,5-triazine: synthesis and biological activity evaluation. Molecules 21:825. 10.3390/molecules21070825 27347912 PMC6273983

[B23] Rosenfeld J. C. (1987). *In-situ* end-capping melt prepared aromatic polyester with phenyl benzoate. US 4680372.

[B24] Ruan Z. X. Huangfu D. S. Xu X. J. Sun P. H. Chen W. M. (2013). 3D-QSAR and molecular docking for the discovery of ketolide derivatives. Expert Opin. Drug Discov. 8, 427–444. 10.1517/17460441.2013.774369 23441865

[B25] Wang F. L. Li C. R. Liu W. X. Wan F. H. (2008). Advance in biological characteristics and control techniques of the new invasive sycamore lace bug (*Corythucha ciliata*). Sci. Silv. Sin. 44, 137–142.

[B26] Wang R. Li F. Q. Zhang W. Zhang X. M. Qu C. Tetreau G.. (2017). Identification and expression profile analysis of odorant binding protein and chemosensory protein genes in *Bemisia tabaci* MED by head transcriptome. PLoS ONE 12:e0171739. 10.1371/journal.pone.0171739 28166541 PMC5293548

[B27] Wang S. S. Sun Y. F. Du S. Q. Qin Y. G. Duan H. X. Yang X. L. (2016). Computer-aided rational design of novel EBF analogues with an aromatic ring. J. Mol. Model. 22, 1–9. 10.1007/s00894-016-3011-3 27251400

[B28] Wanner K. W. Willis L. G. Theilmann D. A. Isman M. B. Feng Q.. (2004). Analysis of the insect os-d-like gene family. J. Chem. Ecol. 30, 889–911. 10.1023/B:JOEC.0000028457.51147.d4 15274438

[B29] Watanabe K. Shimizu N. (2015). Alarm pheromone activity of nymph-specific geraniol in *Chrysanthemum* lace bug *Corythucha marmorata* against adults and nymphs. Nat. Product Commun. 10, 1495–1498. 26594742

[B30] Wu Z. Z. Zhang H. Bin S. Y. Chen L. Han Q. X. Lin J. T. (2016). Antennal and abdominal transcriptomes reveal chemosensory genes in the asian citrus psyllid, *Diaphorina citri*. PLoS ONE 11:e0159372. 10.1371/journal.pone.0159372 27441376 PMC4956155

[B31] Wiederstein M. Sippl M. J. (2007). Prosa-web: interactive web service for the recognition of errors in three-dimensional structures of proteins. Nucleic Acids Res. 35, 407–10. 10.1093/nar/gkm290 17517781 PMC1933241

[B32] Zhou J. J. Vieira F. G. He X. L. Smadja C. Liu R. Rozas J.. (2010). Genome annotation and comparative analyses of the odorant-binding proteins and chemosensory proteins in the pea aphid *Acyrthosiphon pisum*. Insect Mol. Biol. 19, 113–122. 10.1111/j.1365-2583.2009.00919.x 20482644

[B33] Zhou S. S. Sun Z. Ma W. Chen W. Wang M. Q. (2014). *De novo* analysis of the *Nilaparvata lugens* (Stål) antenna transcriptome and expression patterns of olfactory genes. Comp. Biochem. Physiol. Part D Genom. Proteom. 9:31. 10.1016/j.cbd.2013.12.002 24440828

[B34] Zhu J. Iovinella I. Dani F. R. Liu Y. L. Huang L. Q. Liu Y.. (2016a). Conserved chemosensory proteins in the proboscis and eyes of *Lepidoptera*. Int. J. Biol. Sci. 12, 1394–1404. 10.7150/ijbs.16517 27877091 PMC5118785

[B35] Zhu J. Wang G. R. Pelosi P. (2016b). Plant transcriptomes reveal hidden guests. Biochem. Biophys. Res. Commun. 474, 497–502. 10.1016/j.bbrc.2016.04.134 27130825

