# Supplementary figures and images for: Identification of an Alarm Pheromone-Binding Chemosensory Protein From the Invasive Sycamore Lace Bug Corythucha ciliata (Say)
Source: Front Physiol. 2018 Apr 6;9:354. doi: 10.3389/fphys.2018.00354 (PMC5897531; doi:10.3389/fphys.2018.00354)

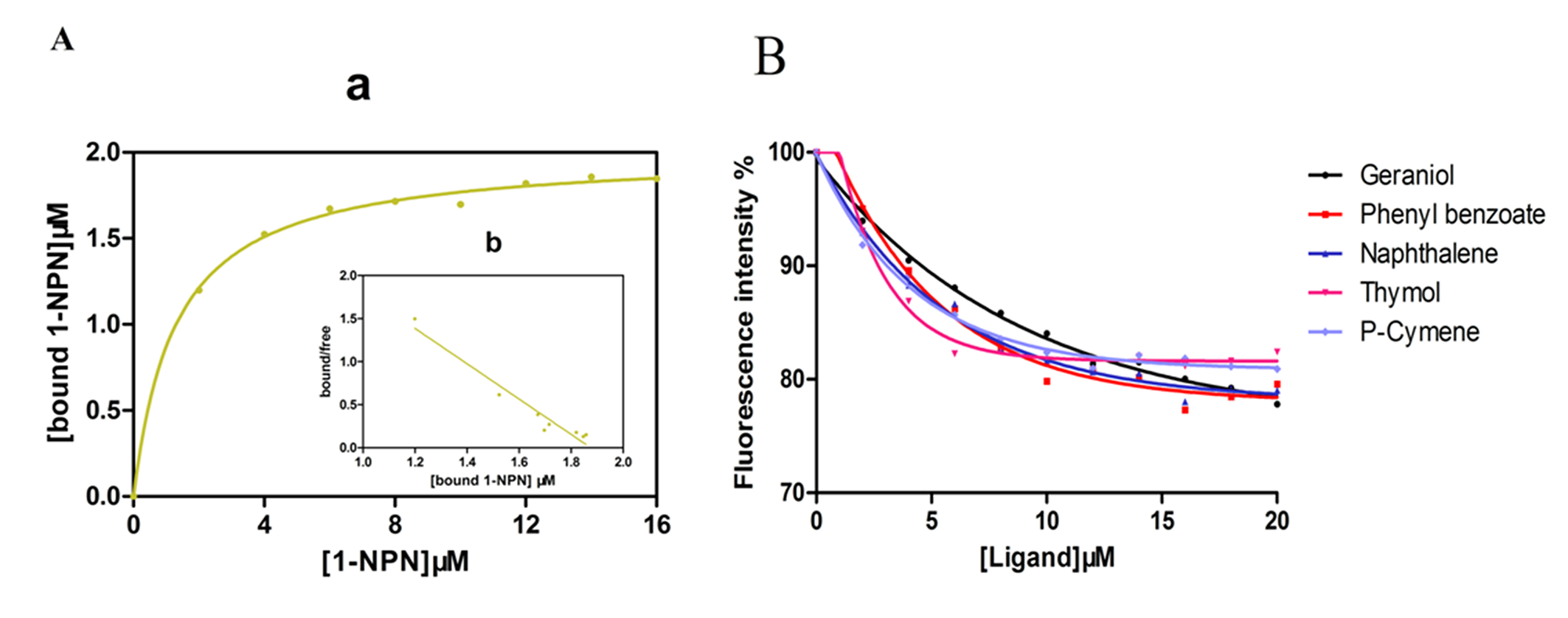

Supplement: Figure S1 — Binding of N-phenyl-1-naphthylamine (1-NPN) and selected ligands to c18915_g1. (A) Affinity of c18915_g1 for 1-NPN. (B) Competitive binding assays. [file Image1.tif]
